# Multiparametric Breast MRI for the Differentiation of Atypical Ductal Hyperplasia and Ductal Carcinoma In Situ in Postmenopausal Women

**DOI:** 10.7759/cureus.101720

**Published:** 2026-01-17

**Authors:** Marko N Mihajlovic, Luka J Raspopovic, Dejan Dimitrijevic, Iva B Krusac, Andjela Djajic, Mirjan M Nadrljanski

**Affiliations:** 1 Department of Diagnostic Radiology, Institute of Oncology and Radiology of Serbia, Belgrade, SRB; 2 Department of Radiology, Faculty of Medicine, University of Belgrade, Belgrade, SRB

**Keywords:** atypical ductal hyperplasia, dce-mri, ductal carcinoma in situ, functional parameters, morpho-dynamic parameters

## Abstract

Introduction

Atypical ductal hyperplasia (ADH) is a proliferative epithelial breast lesion associated with an increased risk of progression to ductal carcinoma in situ (DCIS) or invasive carcinoma. Although breast MRI is highly sensitive for lesion detection, its ability to differentiate biologically related non-mass enhancement (NME) lesions such as ADH and DCIS remains incompletely defined.

Aim

The aim of this exploratory study was to investigate whether predefined morphologic, dynamic, and functional breast MRI parameters, such as lesion size, postcontrast signal intensity increase (wash-in), time-intensity curve (TIC) type, apparent diffusion coefficient (ADC), and positive enhancement integral (PEI), demonstrate measurable differences between ADH and DCIS in postmenopausal women.

Materials and methods

This retrospective, single-center study included 40 postmenopausal women with histopathologically confirmed ADH (n = 20) or DCIS (n = 20) who underwent standardized 1.5-Tesla (T) breast MRI. Quantitative MRI parameters evaluated included lesion size, early postcontrast signal intensity increase (wash-in), time-intensity curve (TIC) type, apparent diffusion coefficient (ADC), and positive enhancement integral (PEI). Groups were matched for age and lesion size. Statistical comparisons were performed using appropriate parametric tests, with a two-sided significance threshold of p < 0.05.

Results

No statistically significant differences were observed between ADH and DCIS across the evaluated MRI parameters, including lesion size (p = 0.119), wash-in (p = 0.484), ADC (p = 0.141), or PEI (p = 0.360). TIC Types 1 and 2 were similarly distributed between groups (p = 0.342), with no lesions demonstrating a washout pattern.

Conclusion

In this exploratory cohort of postmenopausal women, ADH and DCIS demonstrated substantial overlap in morphologic, dynamic, and functional breast MRI parameters. While no statistically significant differences were identified, these findings should be interpreted cautiously given the limited sample size and potential for Type II error. Larger, adequately powered studies incorporating advanced quantitative imaging techniques are required to determine whether multiparametric MRI can reliably differentiate between these entities.

## Introduction

Breast cancer is the most frequently diagnosed malignancy among women worldwide, according to the World Health Organization (WHO) [1]. Its incidence increases with age, with a notable rise after the fifth decade of life [2,3]. The disease predominantly originates from the epithelial components of the breast. Among benign epithelial proliferative lesions, atypical ductal hyperplasia (ADH) is characterized by a clonal proliferation of ductal epithelial cells with cytologic and architectural features resembling low-grade ductal carcinoma in situ (DCIS). Although ADH is not malignant, it is well recognized as a marker of increased breast cancer risk [4,5].

DCIS represents a heterogeneous group of pre-invasive breast lesions defined by the proliferation of malignant epithelial cells confined to the ductal-lobular system without evidence of basement membrane invasion [6]. The diagnosis of DCIS is based on an integrated assessment of histopathological, clinical, and imaging findings. Accurate differentiation between ADH and DCIS is clinically relevant, as it has important implications for patient management, surveillance strategies, and treatment planning.

In routine clinical practice, breast imaging typically follows a standardized diagnostic pathway, beginning with mammography or digital breast tomosynthesis, followed by breast MRI when additional characterization is required. While mammography remains highly sensitive for the detection of microcalcifications, breast MRI offers superior sensitivity for non-calcified lesions, identifying approximately 10%-15% of additional lesions that may be occult on mammography [7,8].

Dynamic contrast-enhanced MRI (DCE-MRI) enables the assessment of both morphologic and functional characteristics of breast lesions by evaluating tumor microvascularization [8]. Malignant and premalignant lesions commonly demonstrate neoangiogenesis, leading to increased vascular permeability. Following administration of gadolinium-based contrast agents, these vascular changes result in rapid contrast uptake and characteristic enhancement patterns, which can be analyzed using time-intensity curves (TICs) to assess wash-in and wash-out kinetics [7-9]. This approach allows for a reproducible evaluation of lesion enhancement behavior and morphology [10].

In addition to DCE-MRI, diffusion-weighted imaging (DWI) and quantitative apparent diffusion coefficient (ADC) measurements provide insight into tissue cellularity and microstructural characteristics. Functional parameters such as ADC and the positive enhancement integral (PEI) have been shown to improve the specificity of breast MRI when used alongside morpho-dynamic analysis [9,10].

The aim of this exploratory study was to investigate whether predefined morphologic, dynamic, and functional MRI parameters, including lesion size, enhancement kinetics, TIC characteristics, ADC, and PEI, show measurable differences between ADH and DCIS in postmenopausal women. The study was designed to generate preliminary data addressing the limited evidence on quantitative MRI differentiation between these biologically related entities.

This study was previously presented in abstract form at the “Mini Simpozijum” conference, Faculty of Medicine, University of Belgrade in Belgrade, Serbia, held from April 19-23, 2021.

## Materials and methods

Study design and patient population

This retrospective, single-center study included postmenopausal female patients who presented with mammographically suspicious breast lesions detected on either two-dimensional full-field digital mammography (FFDM) or digital breast tomosynthesis (DBT), with further evaluation by breast ultrasound (US) and breast MRI.

Inclusion criteria were as follows: (1) breast lesions detectable on both morphologic and functional MRI sequences and (2) histopathological confirmation obtained by image-guided biopsy. Exclusion criteria included the presence of invasive carcinoma, incomplete imaging examinations, surgical biopsy without imaging guidance, and significant artifacts on breast MRI sequences (both morphologic and functional).

An institutional database search initially identified 94 patients with histologically confirmed ADH or DCIS who had undergone a breast MRI. After applying exclusion criteria, 28 patients who underwent surgical or unguided Tru-Cut biopsy for palpable lesions, nine patients with significant artifacts on DWI, and 18 patients with DCIS containing invasive components, a total of 40 postmenopausal women, were included in the final analysis.

Patients were divided into two subgroups according to histopathological diagnosis: subgroup 1 (ADH; n = 20; mean age 58.6 years) and subgroup 2 (DCIS; n = 20; mean age 59.1 years).

The study was conducted between October 2019 and April 2021 and was approved by the Institutional Review Board of the Institute of Oncology and Radiology of Serbia, Belgrade, Serbia, which waived the requirement for written informed consent due to the retrospective nature of the study (approval number: 2044/12052010).

Imaging and biopsy procedures

All patients initially underwent FFDM or DBT. Lesions were categorized according to the Breast Imaging Reporting and Data System (BI-RADS) as categories 4a, 4b, or 4c, warranting tissue sampling. Additional imaging included breast US and DCE-MRI.

Image-guided biopsy was performed using one of the following techniques: MRI-guided vacuum-assisted biopsy (MR-VAB), stereotactic vacuum-assisted biopsy (SVAB), DBT-guided vacuum-assisted biopsy (DBT-VAB), or US-guided core-needle biopsy (CNB). Biopsies were performed at least 14 days prior to MRI examination. Histopathological analysis was conducted using standard diagnostic criteria.

MRI protocol

All MRI examinations were performed using a 1.5-Tesla (T) system (Magnetom Avanto Fit; Siemens Healthineers, Erlangen, Germany) with a dedicated breast coil and a standardized full diagnostic protocol. The protocol included turbo inversion recovery magnitude (TIRM), T2-weighted turbo spin-echo (T2W-TSE), T1-weighted turbo spin-echo (T1W-TSE), and DWI with b-values of 50 and 850 s/mm².

DCE-MRI consisted of four post-contrast three-dimensional fast low-angle shot (FLASH) acquisitions obtained at 1.23-minute intervals. Post-processing included image subtraction, three-dimensional reconstruction, and maximum intensity projection (MIP).

Gadobutrol (Gadovist; Bayer Pharma, Berlin, Germany) was administered intravenously at a dose of 0.1 mmol/kg body weight using an automated injector (Ulrich Medical, Ulm, Germany) at a flow rate of 2 mL/s, followed by a saline flush.

Image analysis was performed on dedicated workstations (Leonardo; Siemens Healthineers) using Syngo (Siemens Healthineers) and OsiriX (Pixmeo, Geneva, Switzerland) software.

MRI data analysis

Morphologic and dynamic lesion characteristics were evaluated based on lesion size and TIC analysis. TICs were generated by assessing the wash-in phase and changes in signal intensity over time within manually placed regions of interest (ROIs) corresponding to the most enhancing portions of each lesion [11-14].

DWI was performed using an echo-planar imaging sequence with b-values of 50 and 850 s/mm². ADC values were calculated using the following formula:

$\mathrm{ADC} = \ln\left(\frac{S_1}{S_2}\right) \big/ (b_2 - b_1)$

where S_1_ and S_2_​ represent the signal intensities obtained at b-values of b1=50 s/mm² and b2=850 s/mm², respectively [9,10]. ADC measurements were derived from manually defined ROIs on the corresponding ADC maps.

The PEI was calculated as the cumulative signal intensity increase above baseline within the selected ROIs, obtained from PEI maps generated during DCE-MRI analysis [15].

Statistical analysis

Statistical analysis was performed using IBM SPSS software (version 21.0; IBM Corp., Armonk, NY, USA). Continuous variables were expressed as mean ± standard deviation and assessed for normality prior to analysis. Between-group comparisons of continuous variables were performed using the independent-samples Student’s t-test. Categorical variables, including TIC types, were compared using the chi-square (χ²) test.

A two-sided p-value < 0.05 was considered statistically significant. No formal a priori or post-hoc power analysis was performed; therefore, the study may have been underpowered to detect small-to-moderate differences between groups, and the absence of statistically significant findings should be interpreted with caution.

The statistical analysis was intended to provide descriptive and exploratory comparisons between ADH and DCIS rather than to establish definitive diagnostic performance.

## Results

Both postmenopausal subgroups, patients with ADH (n1) and DCIS (n2), were matched for age and lesion size. The mean age did not differ significantly between the groups (58.6 ± 5.5 years in the ADH group vs. 59.1 ± 7.2 years in the DCIS group; p = 0.857). Similarly, mean lesion size was comparable between groups (1.50 ± 0.02 cm in ADH vs. 1.60 ± 0.10 cm in DCIS; p = 0.119). Following contrast administration, all lesions in both subgroups demonstrated heterogeneous enhancement patterns.

DCE-MRI analysis focused on wash-in characteristics, defined as the percentage increase in signal intensity within the first 90 seconds after contrast injection. A rapid wash-in (≥90% increase within 90 seconds) was observed in all patients, consistent with prior reports [11,13,14]. Both ADH and DCIS lesions showed similar rapid enhancement trends, reflecting their shared pathological basis as proliferative epithelial lesions.

Functional MRI parameters, including the ADC (×10⁻³ mm²/s) and PEI, were compared between groups using a two-tailed Student’s t-test (significance level p < 0.05). No statistically significant differences were observed between ADH and DCIS for ADC values (p = 0.141) or PEI values (p = 0.360). These findings are consistent with overlapping cellularity and perfusion characteristics in ADH and DCIS [9,12,15]. A summary of demographic, morphologic, dynamic, and functional parameters is provided in Table 1.

**Table 1:** Comparison of clinical and MRI parameters between ADH and DCIS Abbreviations: ADH, atypical ductal hyperplasia; DCIS, ductal carcinoma in situ; ADC, apparent diffusion coefficient; PEI, positive enhancement integral. All comparisons were performed using a two-tailed Student’s t-test (p < 0.05 considered statistically significant).

Variable	ADH (n1=20)	DCIS (n2=20)	t-value	p-value
Age (years)	58.6 +/- 0.05	59.1 +/- 7.2	-0.182	0.857
Lesion size (cm)	1.50 +/- 0.02	1.60 +/- 0.10	-1.596	0.119
Wash-in (%/90 s)	177.3 +/- 41.1	166.3 +/- 56.4	0.708	0.484
ADC (10^-3^mm^2^/s)	1.37 +/- 0.05	1.35 +/- 0.05	1.504	0.141
PEI value	581.86 +/- 73.59	600.57 +/- 52.38	-0.926	0.360

TIC analysis

Delayed-phase dynamic analysis demonstrated either persistent enhancement (TIC Type 1) or a plateau pattern (TIC Type 2) in both subgroups, defined by a maximal signal intensity variation of ±10% [11,13,14]. These kinetic patterns are characteristic of proliferative epithelial and in situ lesions.

The distribution of TIC types between the ADH and DCIS groups was compared using the χ² test. No statistically significant difference was observed (p = 0.342), although TIC Type 2 was slightly more frequent in the DCIS group. These results are summarized in Table 2.

**Table 2:** Distribution of TIC types in ADH and DCIS Comparisons were performed using the chi-square (χ²) test; p < 0.05 was considered statistically significant. Abbreviations: ADH, atypical ductal hyperplasia; DCIS, ductal carcinoma in situ; TIC, time–intensity curve.

	ADH (n1=20)	DCIS (n2=20)	χ^2^ statistic	p-value
TIC Type 1	11	8	0.902	0.342
TIC Type 2	9	12		

The χ² value of 0.902 indicates minimal differences in TIC distribution between groups, suggesting similar vascular and perfusion characteristics in ADH and DCIS. These findings indicate that TIC patterns alone may be insufficient for reliable differentiation between the two entities on breast MRI.

Diagnostic performance of functional parameters

An ADC cutoff value of ≤1.35 × 10⁻³ mm²/s yielded a sensitivity and specificity of 75% in this cohort; however, this finding should be interpreted cautiously and is intended for descriptive purposes only rather than diagnostic validation.

**Figure 1:**
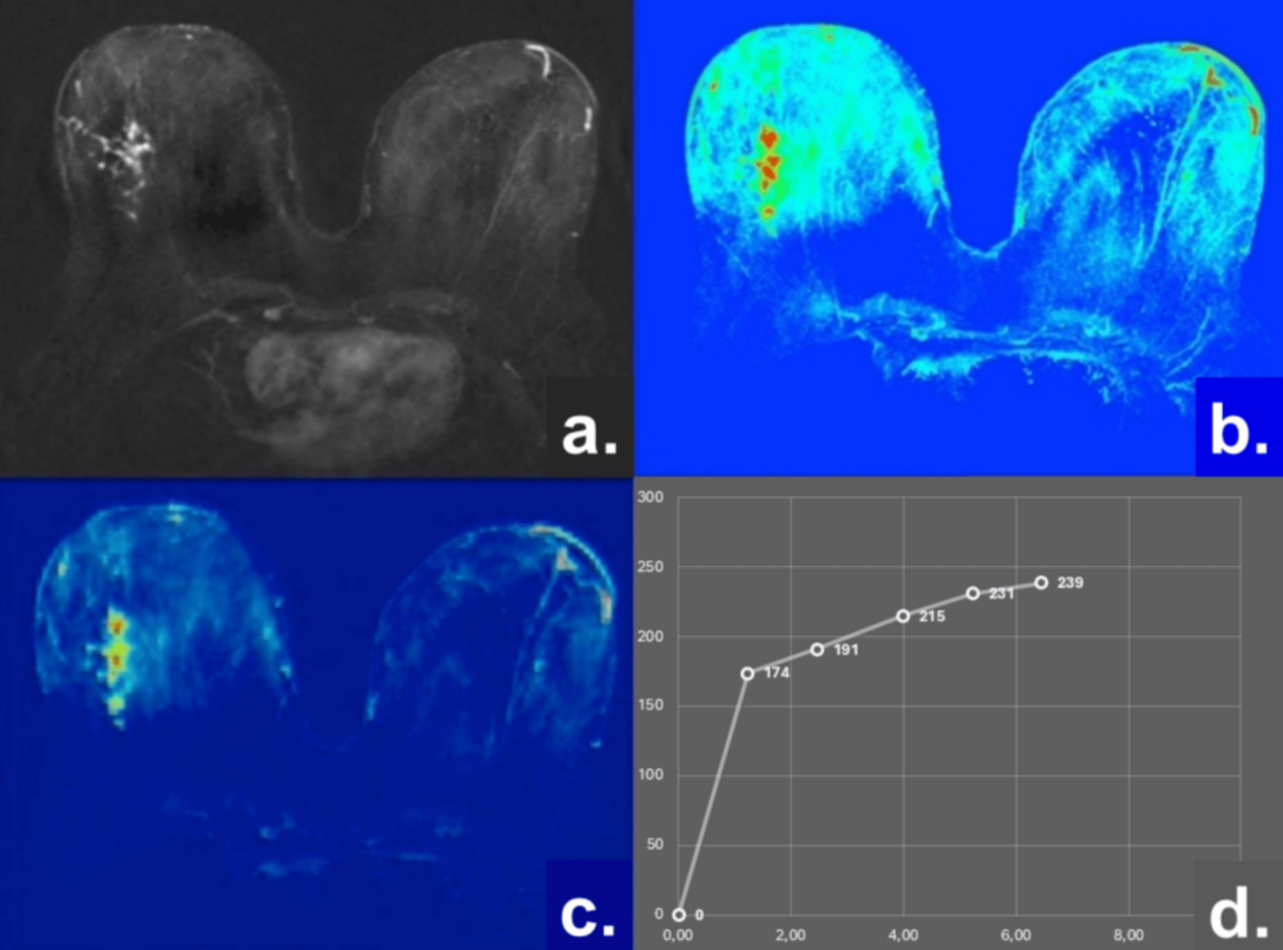
Representative multiparametric breast MRI findings in a patient with ADH (a) Subtraction image demonstrating non-mass enhancement in the right breast; (b) ADC map (1.35 × 10⁻³ mm²/s); (c) PEI = 590.4 ± 20.6; (d) TIC showing a persistent enhancement pattern (TIC Type 1). Abbreviations: ADH, atypical ductal hyperplasia; NME, non-mass enhancement; ADC, apparent diffusion coefficient; PEI, positive enhancement integral; TIC, time–intensity curve.

The results are graphically presented and analyzed in patients with ADH (n1) using a multiparametric MRI approach that includes morphologic assessment, ADC, PEI, and TIC analysis, as illustrated in Figure 1.

**Figure 2:**
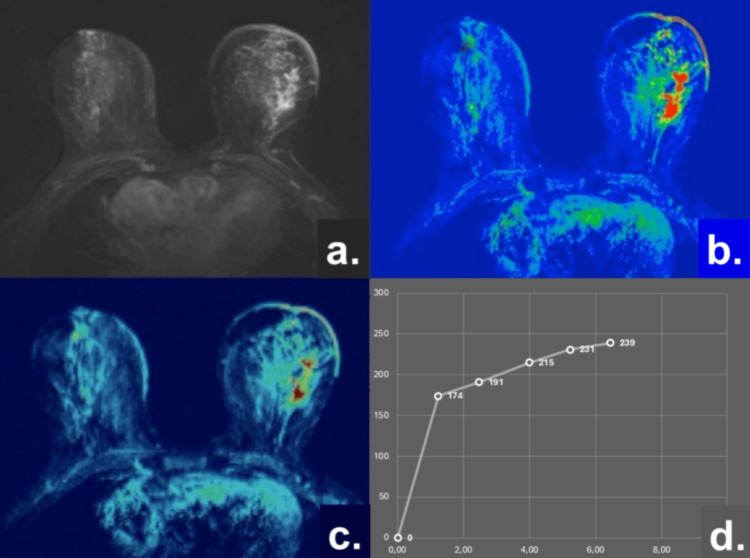
Representative multiparametric breast MRI findings in a patient with DCIS. (a) Subtraction image demonstrating NME in the left breast; (b) ADC map (1.25 × 10⁻³ mm²/s); (c) PEI = 688.4 ± 24.4; (d) TIC showing a persistent enhancement pattern (TIC Type 1). Abbreviations: DCIS, ductal carcinoma in situ; NME, non-mass enhancement; ADC, apparent diffusion coefficient; PEI, positive enhancement integral; TIC, time–intensity curve

Similarly, the results are graphically presented and analyzed in patients with DCIS (n2) using the same multiparametric MRI algorithm, including morphologic assessment, ADC, PEI, and TIC analysis, as illustrated in Figure 2.

## Discussion

The dynamic properties of MRI-detectable breast lesions primarily depend on vascularization and contrast agent pharmacokinetics. Malignant lesions larger than approximately 2 mm are known to secrete angiogenic factors that stimulate angiogenesis and neoangiogenesis, thereby facilitating lesion visualization following contrast administration on MRI [10-12,16]. In such lesions, signal intensity typically increases earlier and more intensely compared with benign lesions. However, ADH and DCIS do not represent classic examples of either benign or malignant pathology. Instead, both entities demonstrate heterogeneous enhancement patterns related to their shared underlying process of neoangiogenesis [10,11,16,17]. The multiparametric approach in this study refers to parallel evaluation of individual MRI parameters rather than the construction of a composite predictive model.

In the present study, all lesions demonstrated a rapid initial increase in signal intensity, which is consistent with their pathohistological characteristics. As expected, analysis of the wash-in parameter revealed no statistically significant difference between the ADH and DCIS groups, indicating similar early enhancement behavior in these proliferative epithelial lesions.

Previous studies have reported inconsistent findings regarding delayed-phase enhancement patterns in DCIS. Mennell et al. described no consistent TIC pattern in DCIS during delayed postcontrast imaging, in agreement with our results [18]. Conversely, other studies have reported a predominance of TIC Type 2 (plateau pattern) in DCIS [10-12,19]. In our cohort, TIC Types 1 and 2 were similarly distributed between ADH and DCIS, which may reflect their shared histopathological features. Furthermore, both ADH and DCIS typically present as non-mass enhancement (NME) lesions, which likely contributes to TIC heterogeneity [11,12,20].

The evaluation of TIC patterns in NME lesions is technically challenging. ROIs in NME often include pixels corresponding to lesions at different stages of development as well as adjacent normal parenchyma, unlike solid masses, where ROIs can be more precisely confined [9,11,17]. These limitations may partly explain the overlap in TIC patterns observed between ADH and DCIS. Although Yang et al. reported significant differences in TIC patterns between benign and malignant lesions [21], the diagnostic value of TIC analysis in NME lesions may be limited and should primarily be interpreted in conjunction with morphologic features. Notably, no lesion in our study exhibited a TIC Type 3 (washout) pattern, further supporting the in situ nature of both ADH and DCIS.

DWI, which evaluates diffusion restriction in highly cellular tissues, has been shown to increase the specificity of breast MRI and reduce unnecessary biopsies [22-25]. The ADC serves as a quantitative biomarker of tissue cellularity and has been widely used to differentiate benign from malignant breast lesions [26-29]. In our study, ADC values did not differ significantly between ADH and DCIS. Similar findings were reported by Imamura et al., who observed no significant ADC differences among histologically distinct NME lesions [20]. These results may be explained by the biological continuum between ADH and DCIS, which share overlapping histological and cellular characteristics.

Importantly, ADC values in both groups (1.37 ± 0.05 × 10⁻³ mm²/s for ADH and 1.35 ± 0.05 × 10⁻³ mm²/s for DCIS) were higher than the commonly accepted cutoff value for malignancy (<1.2 × 10⁻³ mm²/s), in accordance with previously published data indicating higher ADC values in in situ lesions [20,26-30]. Variability in ADC measurements across studies may be attributed to technical factors such as b-value selection and the timing of contrast administration, which can influence ADC standardization.

Compared with mammography, DCE-MRI demonstrates superior sensitivity and accuracy in assessing the extent of NME lesions, including ADH and DCIS. This advantage is largely due to the dependence of lesion visualization on periductal and stromal vascularity [4,6]. Perfusion-related parameters, such as the PEI, provide additional functional information beyond morphologic and kinetic features and may assist in differentiating invasive from in situ lesions [9,15,16].

PEI reflects tissue perfusion within the selected ROI and is influenced by tumor vascularity, vessel density, and permeability [16]. As heterogeneous NME lesions, both ADH and DCIS typically demonstrate variable intralesional signal intensity and heterogeneous texture [16]. Consistent with this, no statistically significant difference in PEI values was observed between the two groups, and the values obtained for DCIS were within the reported range in the literature [9].

This study has several limitations. First, the relatively small sample size and retrospective, single-center design limit statistical power and increase the possibility of a Type II error, meaning that true differences between ADH and DCIS may not have been detected. Consequently, the absence of statistically significant differences should be interpreted with caution and does not exclude the potential diagnostic value of multiparametric MRI in larger cohorts. No formal a priori or post-hoc power analysis was performed; therefore, the study may have been underpowered to detect small-to-moderate effect sizes between groups.

Second, although a standardized MRI protocol and single scanner were used to minimize technical variability, quantitative analysis relied on manually placed ROI, which introduces potential observer-related and ROI-selection bias, particularly in heterogeneous NME lesions. Formal interobserver variability analysis was not performed and represents an additional limitation.

Third, histopathological diagnosis was based on image-guided biopsy, which may be subject to sampling limitations, especially in spatially heterogeneous lesions. Finally, the study population was restricted to postmenopausal women to avoid hormonal confounding, which may limit generalizability to premenopausal populations. 

Despite these limitations, the study provides preliminary, hypothesis-generating data that may inform the design of future, adequately powered prospective studies.

## Conclusions

In this exploratory cohort of postmenopausal women, ADH and DCIS demonstrated substantial overlap in morphologic, dynamic, and functional breast MRI parameters, including enhancement kinetics, ADC, and PEI values. No statistically significant differences were identified; however, these findings must be interpreted cautiously given the limited sample size and the associated risk of Type II error. Rather than indicating definitive limitations of multiparametric MRI, the present results suggest that currently available MRI parameters may be insufficiently discriminatory between ADH and DCIS within small, matched cohorts, reflecting their close biological relationship along a continuum of breast carcinogenesis.

While DCE-MRI remains highly sensitive for detecting lesions requiring biopsy, larger, adequately powered studies incorporating advanced quantitative techniques, such as texture analysis, radiomics, and artificial intelligence-based models, are required to determine whether more robust imaging biomarkers can improve differentiation between ADH and DCIS and refine clinical risk stratification.

## References

[1] (2024). Global breast cancer initiative implementation framework: assessing, strengthening and scaling up of services for the early detection and management of breast cancer. https://www.who.int/publications/i/item/9789240065987.

[2] Hayes J, Richardson A, Frampton C (2013). Population attributable risks for modifiable lifestyle factors and breast cancer in New Zealand women. Intern Med J.

[3] Moulder S, Hortobagyi GN (2008). Advances in the treatment of breast cancer. Clin Pharmacol Ther.

[4] Sattar HA (2013). Female genital system and breast. Robbins Basic Pathology, Ninth Edition.

[5] Tozbikian G, Brogi E, Vallejo CE (2017). Atypical ductal hyperplasia bordering on ductal carcinoma. Int J Surg Pathol.

[6] Bane A (2013). Ductal carcinoma in situ: what the pathologist needs to know and why. Int J Breast Cancer.

[7] Radhakrishna S, Agarwal S, Parikh PM (2018). Role of magnetic resonance imaging in breast cancer management. South Asian J Cancer.

[8] Padhani AR (2002). Dynamic contrast-enhanced MRI in clinical oncology: current status and future directions. J Magn Reson Imaging.

[9] Nadrljanski M, Maksimović R, Plešinac-Karapandžić V, Nikitović M, Marković-Vasiljković B, Milošević Z (2014). Positive enhancement integral values in dynamic contrast enhanced magnetic resonance imaging of breast carcinoma: ductal carcinoma in situ vs. invasive ductal carcinoma. Eur J Radiol.

[10] Nadrljanski M, Milosević Z, Plesinac-Karapandzić V, Goldner B (2013). The role of breast magnetic resonance imaging in the diagnosis of ductal carcinoma in situ (Article in Serbian). Srp Arh Celok Lek.

[11] Kuhl CK, Schrading S, Bieling HB (2007). MRI for diagnosis of pure ductal carcinoma in situ: a prospective observational study. Lancet.

[12] Nadrljanski MM, Milosevic ZC (2020). Relative apparent diffusion coefficient (rADC) in breast lesions of uncertain malignant potential (B3 lesions) and pathologically proven breast carcinoma (B5 lesions) following breast biopsy. Eur J Radiol.

[13] Greenwood HI, Heller SL, Kim S, Sigmund EE, Shaylor SD, Moy L (2013). Ductal carcinoma in situ of the breasts: review of MR imaging features. Radiographics.

[14] Kuhl CK (2007). Current status of breast MR imaging. Part 2. Clinical applications. Radiology.

[15] Alicioglu B, Guler O, Bulakbasi N, Akpinar S, Tosun O, Comunoglu C (2013). Utility of semiquantitative parameters to differentiate benign and malignant focal hepatic lesions. Clin Imaging.

[16] Raza S, Vallejo M, Chikarmane SA, Birdwell RL (2008). Pure ductal carcinoma in situ: a range of MRI features. AJR Am J Roentgenol.

[17] Kuhl CK, Schild HH (2000). Dynamic image interpretation of MRI of the breast. J Magn Reson Imaging.

[18] Menell JH, Morris EA, Dershaw DD, Abramson AF, Brogi E, Liberman L (2005). Determination of the presence and extent of pure ductal carcinoma in situ by mammography and magnetic resonance imaging. Breast J.

[19] Jansen SA, Newstead GM, Abe H, Shimauchi A, Schmidt RA, Karczmar GS (2007). Pure ductal carcinoma in situ: kinetic and morphologic MR characteristics compared with mammographic appearance and nuclear grade. Radiology.

[20] Imamura T, Isomoto I, Sueyoshi E (2010). Diagnostic performance of ADC for non-mass-like breast lesions on MR imaging. Magn Reson Med Sci.

[21] Yang QX, Ji X, Feng LL, Zheng L, Zhou XQ, Wu Q, Chen X (2017). Significant MRI indicators of malignancy for breast non-mass enhancement. J Xray Sci Technol.

[22] Woodhams R, Ramadan S, Stanwell P (2011). Diffusion-weighted imaging of the breast: principles and clinical applications. Radiographics.

[23] Padhani AR, Liu G, Koh DM (2009). Diffusion-weighted magnetic resonance imaging as a cancer biomarker: consensus and recommendations. Neoplasia.

[24] Bonekamp S, Corona-Villalobos CP, Kamel IR (2012). Oncologic applications of diffusion-weighted MRI in the body. J Magn Reson Imaging.

[25] Yoshikawa MI, Ohsumi S, Sugata S (2008). Relation between cancer cellularity and apparent diffusion coefficient values using diffusion-weighted magnetic resonance imaging in breast cancer. Radiat Med.

[26] Yabuuchi H, Matsuo Y, Okafuji T (2008). Enhanced mass on contrast-enhanced breast MR imaging: lesion characterization using combination of dynamic contrast-enhanced and diffusion-weighted MR images. J Magn Reson Imaging.

[27] Partridge SC, Mullins CD, Kurland BF, Allain MD, DeMartini WB, Eby PR, Lehman CD (2010). Apparent diffusion coefficient values for discriminating benign and malignant breast MRI lesions: effects of lesion type and size. AJR Am J Roentgenol.

[28] Costantini M, Belli P, Rinaldi P (2010). Diffusion-weighted imaging in breast cancer: relationship between apparent diffusion coefficient and tumour aggressiveness. Clin Radiol.

[29] Surov A, Meyer HJ, Wienke A (2019). Can apparent diffusion coefficient (ADC) distinguish breast cancer from benign breast findings? A meta-analysis based on 13 847 lesions. BMC Cancer.

[30] Kim KW, Kuzmiak CM, Kim YJ, Seo JY, Jung HK, Lee MS (2018). Diagnostic usefulness of combination of diffusion-weighted imaging and T2WI, including apparent diffusion coefficient in breast lesions: assessment of histologic grade. Acad Radiol.

